# Robotics versus laparoscopy - an experimental study of the transfer effect in maiden users

**DOI:** 10.1186/1750-1164-4-3

**Published:** 2010-04-06

**Authors:** Magnus Anderberg, Johan Larsson, Christina C Kockum, Einar Arnbjörnsson

**Affiliations:** 1 Department of Paediatric Surgery, Children's Hospital Lund, Skåne University Hospital and Lund University, Lund, Sweden

## Abstract

**Background:**

Robot-assisted laparoscopy (RL) is used in a wide range of operative interventions, but the advantage of this technique over conventional laparoscopy (CL) remains unclear. Studies comparing RL and CL are scarce. The present study was performed to test the hypothesis that maiden users master surgical tasks quicker with the robot-assisted laparoscopy technique than with the conventional laparoscopy technique.

**Methods:**

20 subjects, with no prior surgical experience, performed three different surgical tasks in a standardized experimental setting, repeated four times with each of the RL and CL techniques. Speed and accuracy were measured. A cross-over technique was used to eliminate gender bias and the experience gained by carrying out the first part of the study.

**Results:**

The task "tie a knot" was performed faster with the RL technique than with CL. Furthermore, shorter operating times were observed when changing from CL to RL. There were no time differences for the tasks of grabbing the needle and continuous suturing between the two operating techniques. Gender did not influence the results.

**Conclusion:**

The more advanced task of tying a knot was performed faster using the RL technique than with CL. Simpler surgical interventions were performed equally fast with either technique. Technical skills acquired during the use of CL were transferred to the RL technique. The lack of tactile feedback in RL seemed to matter. There were no differences between males and females.

## Background

Conventional laparoscopic (CL) surgery may offer great advantages to patients but can be demanding for the surgeon because of several technical drawbacks. These limitations include 2-dimensional vision with less than optimal perception of depth, disturbance of the eye-hand-target axis, the fulcrum effect, rigid instruments with limited degrees of freedom and limited tactile feedback. These factors might attribute to the relatively long training period required before reaching a professional level [1,2].

The da Vinci^® ^surgical system from Intuitive Surgical^® ^(Sunnyvale, CA, USA) has been available since 1998 and is still the only robotic surgical system available on the market approved for performing surgical interventions in humans. Several advantages with robot-assisted laparoscopy (RL) over CL have been identified: 3-dimensional visualization of the operative field with depth perception, additional degrees of freedom and downscaling of instrument movements, restoration of the eye-hand-target axis and enhanced stability, elimination of the fulcrum effect and improved ergonomics for the surgeon.

One stated consequence of these features is that endoscopic surgical skills are more easily mastered and the learning curve is shortened [2-4]. Some authors have succeeded in performing RL for cases they never tried with CL, possibly indicating that RL is considered easier [5].

A definition of the learning curve can be the amount of practice, in terms of time or number of repetitions, needed to reach a certain level of proficiency for completing a specific task. Parameters used when analysing learning curves are time to complete the task, the number of errors made and actions required. Learning curves in daily practice are often defined by operating time, blood loss, morbidity and length of hospital stay [3]. There is only very scant literature on the comparison of learning curves for RL and CL [3,6]. It is also a challenge to interpret the results of earlier studies, one reason being the different levels of previous experience among the participants. However, to know more about the learning curve in minimally invasive surgery and preferably, as a consequence, to be able to shorten the time needed for operative training before reaching a consistent level, is desirable. We therefore decided to test our hypothesis that surgically maiden subjects perform surgical tasks faster with the robot-assisted laparoscopy technique than with the conventional laparoscopy technique.

## Materials and methods

A power calculation, comparing two groups regarding proportions of the specific trait we wanted to study, was performed. At a given power of 80%, an alpha level of 0.05, and a proportion of 0.8 among cases, a sample of at least 12 cases and 12 control subjects is needed to meet a significant effect when the proportion among control subject is 0.2. A sample of 10 cases and 10 control subjects undergoing three repeated trials will be sufficient, although these repeated measures are mutually dependent.

From a cohort of approximately 500 medical students at Lund University, volunteers were invited to participate in this project and from these, 20 subjects (10 men and 10 women), were randomly selected. The subjects were between 23 and 30 years old and had no prior practical experience of open surgery, CL or RL. There were no drop-outs or excluded participants.

All the subjects were given the same standardized oral and written information by the trial instructor. The tasks the subjects were supposed to perform were also demonstrated once for each of the methods RL and CL before the trial started. One instructor and three evaluators, all working at the Department of Paediatric Surgery were used. The evaluators registered the subjects' performances in accordance with a predetermined template. The subjects were allowed to ask for guidance and the instructor gave them standardized advice along the way. The subjects were not allowed to observe or communicate with each other and were therefore isolated during the study.

The primary and secondary end points were time and accuracy when performing the simulated surgical tasks using robotic and conventional laparoscopic instruments.

Material and equipment used during the study were: the da Vinci^® ^Surgical System from Intuitive Surgical^®^, a robotic needle holder, a robotic DeBakey^® ^grasper, laparoscopic optics from KARL STORZ^® ^0° E-class 26003 AA, a laparoscopic needle holder 26173 KL and a laparoscopic grasper KARL STORZ^® ^33121, a Skin Pad in Jig^® ^L & T 00131 from: http://www.limbsandthings.com and Vicryl^® ^from Ethicon^®^, Polyglactin 910, 2-0 (3 Ph. Eur.), CP-1 36 mm 1/2 c 70 cm.

The workstation was prepared in a standardized way and the participants were allowed to familiarize themselves with the instruments for two minutes before starting the trial. The thread was 20 cm long for both suturing and tying a knot. Each of the 20 students carried out three tasks, grab the needle in a correct way, place three continuous sutures over a rift in the Skin Pad in Jig^® ^and tie a surgical knot. These tasks were done four times with each of RL and CL. The subjects were divided according to gender and half the males and half the females began with RL and CL, respectively. For each set, time and quality indicators (1-6 below) were recorded, giving us a total of 960 sets of data to analyze.

1. Grab the needle - time in seconds

2. Continuous suturing (3 stitches) - time in seconds

3. Tie a knot (double) - time in seconds

4. Damage to the Skin Pad in Jig^®^? - yes/no

5. Dropped needle? - number of times/set

6. Tearing of the thread? - number of times/set. 

[see Additional file 1 and 2]

Each subject's results from the RL and the CL, respectively, were recorded and mean values were calculated for the groups: RL first, CL first, RL last and CL last. The analysis was carried out using Wilcoxon's signed rank test for paired samples and the Mann-Whitney test of two independent samples before and after the cross-over. The transfer effect of difference in experience based on the RL users' prior CL experience and vice versa, was tested regarding the three surgical tasks using the Mann-Whitney test of two independent samples. Friedman's and Wilcoxon's signed rank test were used for analysis of tries of knot tying and suturing. Male and female participants were compared using the Mann-Whitney signed rank-test for paired samples. SPSS statistical software, version 15.0, was used for analysing the data. P < 0.05 was considered significant. This study was approved by the local regional ethical committee March 24^th ^2009 (Dnr 2009/59).

## Results

**Figure 1:**
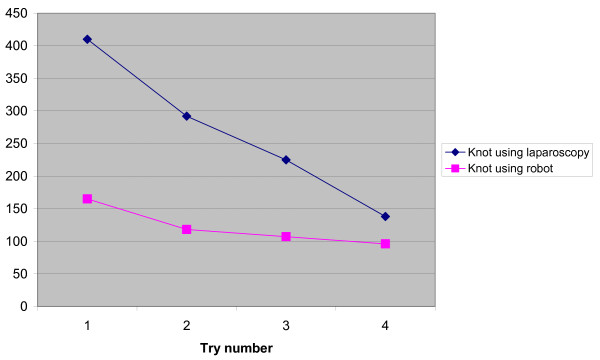
**The mean time for each try to tie a knot for the 20 subjects with CL and RL in seconds**. Statistics: Friedman's test: CL knot, sets 1,2, 3 and 4: 0.0001. RL knot, sets 1,2,3 and 4: 0.3 (ns). Wilcoxon Signed Rank test: CL knot, sets 1 and 4: 0.0001. RL knot, sets 1 and 4: 0.009.

**Figure 2:**
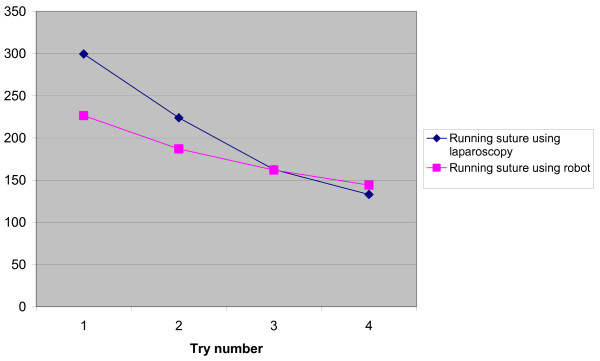
**The mean time for each try of continuous suturing for the 20 subjects with CL and RL in seconds**. Statistics: Friedman's test. CL running suture, sets 1,2,3 and 4: 0,001. RL running suture, sets 1,2,3 and 4: 0,001. Wilcoxon Signed Rank test: CL running suture, sets 1 and 4: 0,001. RL running suture, sets 1 and 4: 0,002.

The values are summarized in Tables 1 and 2. "Grabbing the needle" and "suturing continuously" were carried out at equal speeds with RL and CL. The transfer effect was seen when performing the continuous suturing for RL but not for CL (Tables 3, 4). The same transfer effect was seen for RL in tying a knot but not for CL. The task of tying a knot was performed faster with RL than with CL regardless of whether RL was performed first or second. A difference was observed in tying the knot when changing to the other operating technique, regardless of which technique the subjects started with. The difference favoured RL and was negative for CL (Table 5). Dropping the needle happened more often during the RL part of the study and tearing the thread only occurred with the RL technique. Damage to the Skin Pad in Jig^® ^was equally common (Table 6). There are learning curves seen for tying a knot (Figure 1) and continuous suturing (Figure 2) for both RL and CL when comparing trials 1 and 4. There was no difference between the male and female subjects' performances regarding any of the three tasks or the three quality indicators included in the study.

**Table 1:** Time in seconds for each group of participants.

	Grab the needle	Continuous suturing	Tie a knot
	MEAN ± SD (RANGE)	MEAN ± SD (RANGE)	MEAN ± SD (RANGE)
CL first	6 ± 4 (2 - 25)	198 ± 82 (108 - 472)	266 ± 173 (68 - 935)
RL last	7 ± 5 (2 - 21)	144 ± 70 (57 - 346)	91 ± 35 (45 - 195)
			
RL first	10 ± 8 (1 - 28)	216 ± 123 (76 - 663)	152 ± 94 (48 - 560)
CL last	6 ± 4 (2 - 20)	209 ± 96 (86 - 438)	267 ± 186 (70 - 1027)

N = 10 in each group

**Table 2:** Qualitative parameters for each group of participants.

	Damage	Drop	Break
CL first	1	6	0
RL last	5	32	4
			
RL first	3	47	1
CL last	1	12	0

Number of times damage to pad, dropping the needle or breaking the thread

**Table 3:** Grabbing the needle

	MEAN ± SD (RANGE)	MEAN ± SD (RANGE)	P-value
RL first vs CL first	10 ± 8 (1 - 28)	6 ± 4 (2 - 25)	ns
RL last vs CL last	7 ± 5 (2 - 21)	6 ± 4 (2 - 20)	ns
RL first vs RL last			ns
CL first vs CL last			ns
RL first vs CL last			ns
CL first vs RL last			ns

Time in seconds for each group of participants

**Table 4:** Continuous suturing.

	MEAN ± SD (RANGE)	MEAN ± SD (RANGE)	P-value
RL first vs CL first	216 ± 123 (76 - 663)	198 ± 82 (108 - 472)	ns
RL last vs CL last	144 ± 70 (57 - 346)	209 ± 96 (86 - 438)	ns
*RL first vs RL last*			*0.049*
*CL first vs CL last*			ns
RL first vs CL last			ns
CL first vs RL last			ns

Time in seconds for each group of participants and *transfer effect*

**Table 5:** Tying a knot.

	MEAN ± SD (RANGE)	MEAN ± SD (RANGE)	P-value
RL first vs CL first	152 ± 94 (48 - 560)	266 ± 173 (68 - 935)	0.005
RL last vs CL last	91 ± 35 (45 - 195)	267 ± 186 (70 - 1027)	0.001
*RL first vs RL last*			*0.004*
*CL first vs CL last*			*ns*
RL first vs CL last			0.037
CL first vs RL last			0.005

Time in seconds for each group of participants and *transfer effect*

**Table 6:** Qualitative parameters for all 20 participants after four repeats with RL and CL expressed as the number of times they occurred (n)

	RL	CL	P-value
Damage (n)	8	2	ns
Dropped needle (n)	79	18	< 0.05
Torn thread (n)	5	0	< 0.05

## Discussion

Very few studies comparing the learning curves of RL and CL have been published. In the experimental setting a diversity of parameters, not always well-defined, has been used for analysis of learning curves and only the very beginning of the learning curve is studied. In the clinical setting, an experience bias has been expected due to prior laparoscopic experience of the participating surgeons [2,6-12]. Both experimental and clinical studies show diverging learning curves for robotic surgery. The results of previous studies are not conclusive and to objectively evaluate the learning curve of robotic surgery is difficult.

Our experimental study included participants without any prior experience of open surgery, RL or CL, making the group homogeneous. The performed tasks, well-defined and described, closely mimicked some of the proper surgical procedures used every day in the operating theatre. We used the only robotic surgical system currently on the market and standard CL instruments. The size of our group of participants and the number of repetitions studied was decided after power calculation.

The task of tying a surgical knot was always faster with RL than with CL, even when the participant had gained no experience by carrying out the first part of the study with CL. There are learning curves seen for tying a knot for both RL and CL when comparing trials 1 and 4. The learning curve is steeper for CL but the curves never cross (Figure 1). These findings differ from most previous studies where the initial performance with RL is often inferior to the performance with CL [3]. In a recent publication by Stefanidis et al. the authors reported that robotic assistance significantly improved intracorporeal suturing performance and shortened the learning curve. They also reported that performance of laparoscopic knot tying without robotic assistance did not improve after three repeats [13]. The first statement is supported by our study but the latter is not since we also saw a significant learning curve for CL. Performing more advanced tasks like tying a knot might be faster for maiden users due to the fact that RL is more "intuitive" with instrument movements mimicking normal hand movements. This is supported by some authors [4]. The fact that RL is performed with 3-dimensional vision instead of the 2-dimensional vision in CL might also improve the performance, as has been suggested by others [7,14].

The transfer effect, with a faster performance if the specific method was used as the second part of the study when the tasks had already been tried by the first method, was seen for continuous suturing and tying a knot with RL, but not for CL. This might be interpreted as the RL method being easier to adapt to once acquaintance had been made with the tasks themselves, at least for maiden users. The study by Blavier et al. showed worse performance when shifting from one method to the other in both directions. The shorter learning curve for RL noted by the same authors is supported by our study [7].

The learning curve consists of an initial steep phase in which performance improves rapidly. When the change in improvement slows down, the learning curve reaches a plateau phase in which variability in performance is small. The number of repetitions reported here are too low to reach consistency, which characterizes the end of the learning curve. The learning curve for CL was steeper, but the number of repetitions too few to disclose a complete learning curve. This was not the aim of the study. We concentrated on the first phase of the learning curve in order to detect even small changes or differences between the two techniques used. From our data, we can therefore only conclude that it is initially easier for novice subjects to use robotic assistance for the specific tasks using the set performance parameters. Whether or not the curves for RL and CL eventually cross after more repetitions, or when the plateau phase of the learning curve for each technique is reached, remains unclear. This could be the aim of another study in the future.

The objective structured assessment of technical skill (OSATS) described by Reznick et al. is a validated tool widely used in the education literature. The OSATS is feasible, reliable and can be used for testing technical competence with high clinical relevance [15]. Since we focused on comparing the different repeats and the transfer effect in all three tasks we did not calculate a total score for the time and accuracy parameters.

Dropping the needle was more common in the RL group. Half of the subjects dropped the needle while performing CL and all but two while performing RL. Furthermore, the thread was only torn when using RL. Tactile feedback is not yet possible in RL, which is the most probable explanation for our findings. In spite of these differences, albeit significant, the performance when using RL was not slower in the task "continuous suturing" compared with CL. Without the dropping of the needle in the task "continuous suturing", RL might have been faster. Learning curves are also seen for a continuous suture for both RL and CL when comparing trials 1 and 4 (Figure 2). The two figures 1 and 2 express the mean time for each try for the specific task but do not consider in what order the task is performed. As already stated, no difference was noted between RL and CL for "continuous suturing". Clinical reports have indicated that the improved vision in RL seems to make up for the lack of tactile feedback for more experienced surgeons [5]. The tearing of threads and dropping of needles is probably a greater challenge to the beginner.

The end points of our study, time and accuracy, may not be the best end points to measure. Length of pathway and economy of movement might be better predictors of learning curve and safe performance of laparoscopic surgery. A further possible limitation of our study is that error reduction, an important goal of training, was not measured. The study of Narazaki et al. suggests that both task completion time and distance travelled is shortened with training in novice users [10].

The suggested advantage of faster laparoscopy in the RL group might not be relevant in clinical surgery since inexperienced users are not supposed to perform advanced laparoscopic surgery. Robotic surgeons today are often senior surgeons and already expert laparoscopists. However, the training to become an expert takes a lot of time and is costly, so learning curves are important also for the future education of young surgeons. If RL is proven easier to master with equal or better results than CL, robotic surgery could be an option for efficient surgical training. The many steps of a surgical intervention each have a learning curve and if learning curves are shorter for RL it may have some clinical relevance even at later stages of training.

RL is still in its infancy but offers great opportunities for the future. Major improvements in the availability of tactile feedback and specifically designed instruments are necessary and expected soon. More research needs to be done to define the exact indications for RL to justify the increased costs and the increased time consumption involved, compared with CL.

Whether or not these features with improved accuracy, dexterity and visualization enhance surgical performance remains unclear.

In conclusion, we found support for our hypothesis that a surgical task, such as tying a knot, was performed faster using RL than with CL, while easier surgical tasks could be performed equally fast with either technique. The lack of tactile feedback in RL is a factor to consider at least for maiden users. Experience from one technique was transferred to the other. Our data do not support the suggestion that considerable CL experience is important for those starting to use RL. On the other hand, previous experience did matter in our study. No difference between the performances of male and female subjects was noted.

## Competing interests

When performing this work, there were no external influences or conflicts of interest. None of the authors or subjects received fees from the manufacturers of the material or the instruments used in the study reported here.

## Legal requirements

The authors guarantee that the manuscript will not be published elsewhere in any language without the consent of the copyright owners, that the rights of third parties will not be violated, and that the publisher will not be held legally responsible if there should be any claims for compensation. This study complies with the current laws of the country in which it was performed. This work was performed in accordance with the rules of the ethical committee at our centre and the ethical standards laid down in the 1964 Declaration of Helsinki.

## Authors' contributions

MA designed the study, coordinated all steps of the study, collected and analyzed the data and wrote the paper. JL gathered all participants of the study, collected the data and took active part in writing of the paper. CCK participated in the design of the study, collected the data and took active part in writing of the paper. EA participated in the design of the study, collected and analyzed the data and took active part in writing of the paper. All authors read and approved the final manuscript.

## Supplementary Material

Additional file 1 **The Skin Pad in Jig^®^**. The Skin Pad in Jig^® ^as an *.jpg file, showing the needle, the placed running suture and the tied knot. Click here for file

Additional file 2 **The tasks of this experimental study performed with robot-assisted laparoscopy**. A short video showing the actual tasks in this experimental study performed with robot-assisted laparoscopy. (19 Mb, 130 seconds) Click here for file
