# Supplementary figures and images for: Robotics versus laparoscopy - an experimental study of the transfer effect in maiden users
Source: Ann Surg Innov Res. 2010 Apr 6;4:3. doi: 10.1186/1750-1164-4-3 (PMC2857839; doi:10.1186/1750-1164-4-3)

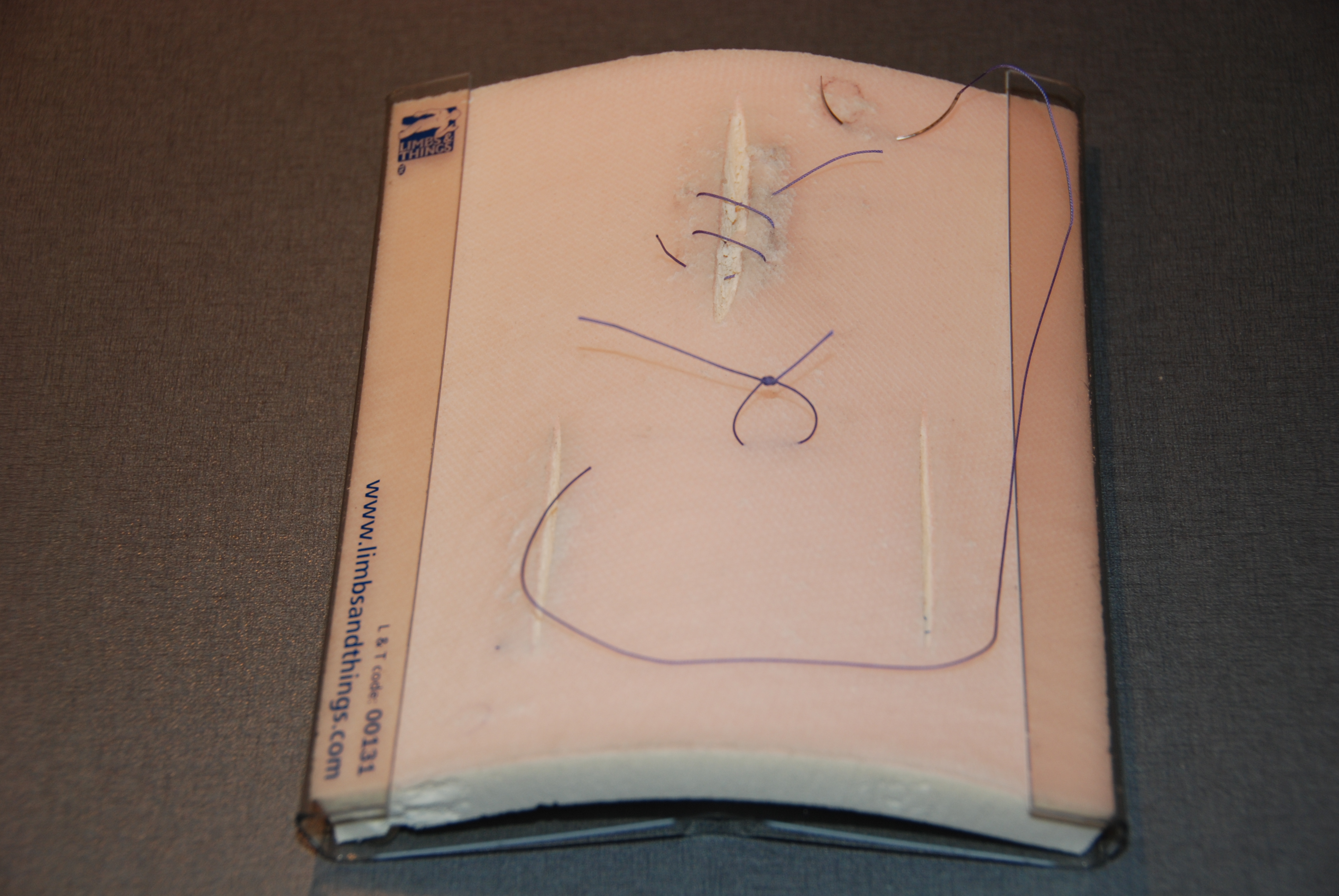

Supplement: Additional file 1 — The Skin Pad in Jig®. The Skin Pad in Jig® as an *.jpg file, showing the needle, the placed running suture and the tied knot. [file 1750-1164-4-3-S1.JPEG]
